# Adipose Tissue as a Strategic Source of Mesenchymal Stem Cells in Bone Regeneration: A Topical Review on the Most Promising Craniomaxillofacial Applications

**DOI:** 10.3390/ijms18102140

**Published:** 2017-10-13

**Authors:** Francesco Paduano, Massimo Marrelli, Massimiliano Amantea, Carlo Rengo, Sandro Rengo, Michel Goldberg, Gianrico Spagnuolo, Marco Tatullo

**Affiliations:** 1 Biomedical Section, Stem Cells Unit, Tecnologica Research Institute, 88900 Crotone, Italy; francesco.paduano@tecnologicasrl.com; 2 Unit of Craniomaxillofacial Surgery, Calabrodental, 88900 Crotone, Italy; research@tecnologicasrl.com; 3 Unit of Experimental Medicine, Marrelli Hospital, 88900 Crotone, Italy; massimiliano.amantea@calabrodental.it; 4 Department of Neurosciences, Reproductive and Odontostomatological Sciences, University of Naples “Federico II”, 80138 Naples, Italy; carlorengo@alice.it (C.R.); sanrengo@unina.it (S.R.); gianrico.spagnuolo@gmail.com (G.S.); 5 Professeur Emerite, Biomédicale des Saints Pères, Université Paris Descartes, Institut National de la Santé et de la Recherche Médicale UMR-S 1124, 75654 Paris, France; michel.goldberg@parisdescartes.fr

**Keywords:** adipose-derived stromal/stem cells (ASCs), craniomaxillofacial bone defects, bone tissue engineering, bone defects, molecular mechanism, clinical trials, surgical reconstruction

## Abstract

Bone regeneration in craniomaxillofacial surgery represents an issue that involves both surgical and aesthetic aspects. The most recent studies on bone tissue engineering involving adipose-derived stromal/stem cells (ASCs) have clearly demonstrated that such cells can play a crucial role in the treatment of craniomaxillofacial defects, given their strong commitment towards the osteogenic phenotype. A deeper knowledge of the molecular mechanisms underlying ASCs is crucial for a correct understanding of the potentialities of ASCs-based therapies in the most complex maxillofacial applications. In this topical review, we analyzed the molecular mechanisms of ASCs related to their support toward angiogenesis and osteogenesis, during bone regeneration. Moreover, we analyzed both case reports and clinical trials reporting the most promising clinical applications of ASCs in the treatment of craniomaxillofacial defects. Our study aimed to report the main molecular and clinical features shown by ASCs, used as a therapeutic support in bone engineering, as compared to the use of conventional autologous and allogeneic bone grafts.

## 1. Introduction

Nowadays, autologous and allogeneic bone grafts still represent the gold standard for repairing bone defects caused by several diseases, congenital defects, infections, and trauma [1,2]. However, bone grafting procedures involve several disadvantages, such as postoperative pain, morbidity at the donor site, infections, and immunological issues, which have prompted the search for a suitable bone substitute that can mimic the osteogenic potential of autogenous bone [3,4].

In this context, mesenchymal stem cells derived from adipose tissue (ASCs) have shown interesting qualities, making them a promising step forward with respect to bone grafts [5,6]. ASCs are able to directly differentiate into mature osteoblasts; moreover, such cells can produce chemokines that are useful for facilitating the homing of endogenous stem cells to the site of bone defect [7,8].

ASCs have interestingly shown the ability to release plasma membrane-derived vesicles (MVs) into the microenvironment: These vesicles are known to act as important mediators in cell-to-cell communication [9]. Thus, ASCs are able to exert a paracrine effect on neighboring cells, whereas extracellular signaling between ASCs and the other differentiated cells is mediated by these MVs. In fact, MVs secreted by ASCs might deliver several types of molecules, such as growth factors, cytokines, RNAs, and microRNAs (miRNA), even to distant locations throughout the body, and perform their biological activities on a number of target cells [9]. Thanks to their paracrine effect, ASCs can induce strong biological effects on target cells, such as promoting cell proliferation and differentiation, as well as the activation of regenerative and reparative processes [10]. ASCs’ paracrine effects can also affect bone regeneration, since MVs are able to deliver osteogenic growth factors, such as bone morphogenetic protein 2 (BMP-2), which may favor local bone tissue regeneration [9].

In the scientific literature, ASCs have successfully been combined with biomaterials, such as β-tricalcium phosphate (β-TCP) [11], bioactive glass (BAG) [12], and platelet-rich plasma (PRP) [13], to improve bone regeneration in animal models: Such studies on animals, together with several case reports on humans, provided interesting results in the field of bone regeneration [14,15,16,17,18,19]. These results describe a new approach to bone tissue regeneration, based on ASCs/scaffold constructs; however, a deep analysis of the studies reported in the literature would be useful to understand the real limits and perspectives of ASCs in bone replacement, from a clinical and molecular point of view.

Although ASCs have been indicated as a promising tool for the treatment of severe bone defects, it has been shown that donor age negatively affects the osteogenic commitment of ASCs, emphasizing a remarkable limitation in their therapeutic potential [20]. ASCs derived from young donors showed a higher expression of osteogenic markers, such as osteopontin (OPN), osteocalcin (OCL), and BMP-2, and a higher content of mineral calcium deposits with respect to elderly patients [20]. Thus, when we are considering using ASCs in bone reconstruction of the craniomaxillofacial region, it is also important to expect the notable influence of donor age on the proliferation and on the osteogenic differentiation of ASCs [20].

The aim of this review is to investigate the potentialities of ASCs-based therapies to treat craniomaxillofacial bone defects. This review will also shed light on the molecular mechanisms, through which ASCs are able to stimulate the main regenerative processes in the treatment of bone defects [21]. A correct understanding of the molecular mechanisms underlying the osteogenic commitment of ASCs would help in the selection and manipulation of such stem cells in a more efficient way and could create new therapeutic strategies to treat bone defects, with special attention to craniomaxillofacial sites.

## 2. Molecular Characteristics of Adipose-Derived Stromal/Stem Cells (ASCs): Gene Expression Profile and Secretome Analysis

Bone healing is a complex process involving several molecular, biochemical, and cellular mechanisms: Among them, angiogenesis plays a particular role in the bone regeneration process [22]. The formation of new blood vessels inside bone defects ensures the critical task of providing those sites with oxygen, nutrients, and growth factors, which facilitate bone formation [23]. Thus, an ideal bone graft should have excellent angiogenetic properties. In this context, ASCs, when compared to other mesenchymal stem cells (MSCs), show a specific ability to secrete growth-factors, inducing angiogenesis, such as vascular endothelial growth factor (VEGF), platelet-derived growth factor (PDGF), and hepatocyte growth factor (HGF), indicating that ASCs are particularly suitable for bone tissue engineering [24]. When compared to bone marrow stromal cells (BMSCs), ASCs show higher proangiogenic activities, mediated by the matrix metalloproteinases MMP-3 and MMP-9 [25]. Moreover, ASCs have been reported to have a high expression of insulin-like growth factor-1 (IGF-1), vascular endothelial growth factor-D (VEGF-D), and interleukin-8 (IL-8), improving the abovementioned proangiogenic commitments of ASCs [26]. Finally, ASCs secrete VEGF that are able to promote new blood vessel formation, and are also able to recruit hematopoietic stem cells [27]. Such molecular characteristics make ASCs one of the most suitable MSCs targeting bone tissue engineering.

ASCs also show the ability to secrete bone morphogenetic protein 2 (BMP-2), which plays an essential role in bone regeneration [28], as well as fibroblast growth factor-2 (FGF-2), keratinocyte growth factor (KGF), and insulin-like growth factor-1 (IGF-1), which are involved in the wound healing process [24].

As described in the Introduction, another important characteristic of ASCs is their ability to secrete membrane-derived microvesicles (MVs) that contain several pro-angiogenic molecules, including FGF2, PDGF, VEGF, MMP2, MMP9, as well as osteogenic molecules, such as BMP2 [9,29].

Thus, MVs secreted by ASCs are able to transfer these growth factors to neighboring cells by exerting pro-angiogenic and osteogenic effects. For this purpose, it has been shown that MVs released from ASCs were able to increase the migration and tube formation of human umbilical vein endothelial cells (HUVECs), indicating that MVs from ASCs promote the angiogenesis of target cells, a process that is required for bone regeneration [10]. Collectively, these data provide evidence that ASCs can actively influence the behavior of target cells through the secretion of MVs [9].

Although several studies have reported a better commitment of BMSCs when compared to ASCs with respect to osteogenic differentiation [30,31,32], the mRNA levels of *BMP-2*, collagen type I (*COL1A1*), and osteonectin (*ON*) were expressed in higher amounts by undifferentiated ASCs than by BMSCs [33]. In addition, it has been found that ASCs express and secrete various growth factors and cytokines, such as receptor activator of nuclear factor κ-B ligand (RANKL), macrophage colony-stimulating factor (M-CSF), BMP-4, and extracellular matrix proteins, including fibronectin (FN) and type I collagen (ColI), which are involved in bone remodeling [21].

Finally, it is important to underline that ASCs can survive in low-oxygen environments, making them optimal for cell therapies where oxygen supplied by the vascular network may be limited, such as in implant surgery procedures [34]. In fact, under hypoxic conditions, ASCs are able to increase VEGF secretion, stimulating the proper angiogenesis [35] needed for bone formation [36].

## 3. Molecular Mechanisms Responsible for Osteogenic Commitment of ASCs

Although ASCs have been extensively used in bone reconstruction, not much is known regarding the molecular mechanisms that regulate the osteogenic commitment of these cells.

Liu et al. reported that the extracellular signal-related kinase (ERK) pathway plays a central role in the osteogenic commitment of ASCs. In fact, the activation of the ERK pathway in ASCs leads to the expression of osteogenesis-related genes, such as *Cbfa1*, *ColI*, *ALP*, and *OCN*, which are responsible for mineralization of the extracellular matrix (ECM). On the contrary, the interruption of the same pathway leads to the inhibition of osteogenic differentiation [37]. Thus, a careful balance between osteogenesis and adipogenesis, occurring in ASCs, seems to be closely regulated by the MAPK/ERK pathway: The phosphorylation of peroxisome proliferator-activated receptor gamma (PPAR-γ), carried out by ERK, decreases its transcriptional activity, resulting in an anti-adipogenic effect [37]. ERK activation also promotes the MAPK-dependent phosphorylation of PPAR-γ, contributing to the switch from adipogenesis to osteogenesis in ASCs.

It has also been shown that the tumor necrosis factor-alpha (TNF-α), an activator of ERK, plays a crucial role in the stronger commitment of ASCs toward the osteogenic lineage [38]. In fact, TNF-α may enhance the osteogenic differentiation of ASCs by increasing specific gene expressions, such as osteopontin (*OPN*), runt-related transcription factor 2 (*RUNX-2*), and alkaline phosphatases (*ALP*) [39]. In the literature, it has been demonstrated that TNF-α induces the osteogenic differentiation of ASCs through the activation of the NF-κB signaling pathway [38]; furthermore, NF-κB increases the expression of the transcriptional co-activator with a PDZ-binding motif (TAZ), which stimulates ASCs into differentiating into osteoblasts through the activation of *RUNX-2* and the repression of *PPAR-γ* transcription. Therefore, osteogenic induction of ASCs has been demonstrated to be mainly mediated by the activation of NF-κB by TNF-α, which promotes the subsequent increase in the expression level of the transcriptional coactivator TAZ [38].

A recent study reported that Notch signaling is also directly involved in the osteogenic differentiation of ASCs [40]. It has been shown that Notch pathway activation improves the osteoinductive ability of ASCs and the formation of the extracellular matrix, whereas the Notch inhibition downregulates the osteogenic commitment of these cells [40]. These studies suggest that the modulation of Notch signaling could be used to stimulate an osteogenic induction of ASCs.

Finally, other pathways, such as p38 MAPK, transforming growth factor beta (TGF-β) [41], and c-Jun N-terminal kinase (JNK) [21], are also closely related to the osteogenic induction of ASCs. An understanding of the molecular mechanisms underlying the osteogenic induction of ASCs could be used to improve bone formation in patients with critical-sized defects in the craniomaxillofacial region.

## 4. Clinical Case Reports: ASCs Combined with Bioscaffolds for Craniomaxillofacial Bone Regeneration

The first clinical report about the use of ASCs to augment bone tissue was described by Lendeckel et al. The authors reconstructed a large post-traumatic calvarial defect by using autologous fibrin glue and a resorbable macroporous sheet [14]; they obtained a stable osteointegrated graft with a good ossification, only three months after the surgery.

The use of ASCs to repair complex craniofacial defects has been well reported in the scientific literature; however, technical variations were mainly related to the biomaterials used as bioscaffolds. Mesimaki et al. [15] used GMP-grade human autologous ASCs, combined with β-tricalcium phosphate (β-TCP) and bone morphogenetic protein-2 (BMP-2) [42], to reconstruct a large maxillary defect resulting from the removal of a keratocyst; ASCs combined with β-TCP and BMP-2 were first implanted into a patient’s muscle to allow for ectopic bone formation and was then transplanted to the palate defect. As a result, the newly-formed bone implant was well integrated into the palatal site. This specific study also highlighted that ASCs are able to stimulate cytokines and chemokines that work as homing signals, attracting endogenous/progenitor stem cells to the surgical site [43]. Mesimaki and colleagues also reported that the ASCs used in the regenerative construct showed elevated expressions of osteogenic-related markers, such as *RUNX-2*, *OC*, *OP*, and *ColI*, confirming that ASCs have an optimal behavior for bone tissue engineering [15].

More recently, the same researchers successfully showed that autologous human-ASCs combined with β-TCP granules were able to repair large cranial defects in four humans [7].

Furthermore, in a preliminary clinical study by Sandor, the use of ASCs, seeded on bioscaffolds combined with BMP-2, was described [16]. These ASCs seeded on resorbable scaffolds, such as BAG and β-TCP, were successfully implanted into 20 of 23 patients with large craniofacial bony defects. These case reports are extremely encouraging, as they indicate that a favorable cocktail of growth factors or cytokines, combined with ASCs and a suitable scaffold, can be used for the reconstruction of craniofacial osseous defects.

Another clinical report demonstrated the use of ASCs to treat a large bone defect at the mandibular symphysis [17]. This case reported methods quite similar to those of Mesimaki et al. [15] (autologous ASCs, β-TCP granules, and BMP-2) for the treatment of bone defects, but without requiring any ectopic bone formation; in this study, implanted ASCs, combined with the described biomaterials, were able to work synergistically to improve bone replacement.

Similar studies from the same group, using an engineered construct consisting of ASCs, β-TCP granules, and human recombinant BMP-2 combined with computer-aided manufacturing (CAM) [18], analyzed the marker profiles expressed by osteoblasts developed from the ASCs construct: These adult cells have a strong expression of mesenchymal markers, including CD105, TWIST-1, and CD90, a moderate expression of fibroblast growth factor receptor 3 (FGFR3) and VEGF, and a low expression of vascular endothelial growth factor receptors 1-2-3 (VEGFR1-2-3), fibroblast growth factor receptor 2 (FGFR2), TGF-β, ALP and bone morphogenetic protein receptor type 1 (BMPR1). These results showed a strong expression of mesenchymal stem cell markers and a moderate to low expression of angiogenic and osteogenic markers [18].

Sandor et al. also performed other clinical studies describing the successful reconstruction of complex craniomaxillofacial defects, using a combination of MSCs derived from autologous adipose tissue with four different scaffolds: Two types of BAG, β-TCP granules, and pliable strips with β-TCP. Furthermore, these authors decided to use ASCs because of their ability to survive in low-oxygen conditions; moreover, ASCs are able to secrete several cytokines, including VEGF and HGF, which contribute to the formation of a vascularized bone graft, a pre-requisite in the treatment of large bone defects [19].

Despite the limited number of patients, all of these case reports (Table 1) indicate that adipose-derived stem cells can provide strong aid to bone regeneration in the craniomaxillofacial district, without any of the risks of traditional bone grafts.

## 5. Clinical Trials: Adipose-Derived Mesenchymal Stem Cells for the Treatment of Craniomaxillofacial Bone Defects

The successful results in the abovementioned clinical case reports encouraged many researchers to carry out more structured clinical trials to assess the effects of ASCs, seeded on custom-made bone implants, used in the reconstruction of craniomaxillofacial bone defects. Several clinical trials reported positive results with the use of adipose-derived stem cells for maxillofacial applications (Table 2).

Therapeutic applications of ASCs in humans have been performed up to phase II clinical trials. We selected two clinical trials that are registered on the official clinical trial website (www.//clinicaltrials.gov) as of 28 July 2017: They investigated the use of adipose-derived stem cells for the regeneration of maxillofacial bone defects, as reported in more details in Table 2. Both of the studies used a bone graft substitute called Bonofill: It was made by seeding human autologous adipose-derived stem cells on scaffolds composed of mineral particles (Oragraft).

The first study, entitled “Filling Bone Defects/Voids With Autologous Bonofill For Maxillofacial Bone Regeneration” (NCT02153268), investigated the safety and efficacy of autologous ASCs combined with Oragraft mineral particles (Bonofill) in the reconstruction of maxillofacial defects, with results reported six months after surgical implantation.

The second study was a phase-I/II clinical study, entitled “Filling Bone Defects/Voids with Autologous BonoFill-II for Maxillofacial Bone Regeneration” (NCT02842619): the study is currently recruiting patients in order to evaluate the safety and efficacy of human adipose-derived stem cells combined with OraGraft mineral particles for large post-oncological reconstructions of maxillofacial bone.

Both of these clinical trials were based on the use of a manufactured bone substitute, called Bonofill, functionalized with ASCs from autologous adipose tissue: These grafts were fully tolerated and did not induce any immune response or rejection.

## 6. Conclusions and Future Perspectives

Bone regeneration in craniomaxillofacial surgery is an issue that involves both surgical and aesthetic aspects. Several mesenchymal stem cells have been used largely in complex or extensive bone defects [44,45,46,47,48]; however, stem cells taken from traditional sites have typically shown some disadvantages, resulting from the difficulty in tissue sampling and from scarce tolerance to harvesting procedures, as reported by patients. ASCs can be obtained through minimally-invasive procedures, in large numbers, from lipoaspirates or surgical resection, with low donor-site morbidity [49], and they can be rapidly expanded, with respect to bone marrow stromal cells (BMSCs) [50,51].

Bone regeneration and repair are complex physiological processes, regulated by different cell types, as well as extracellular matrix proteins and growth factors [18]. In this context, it has been shown that ASCs can secrete growth factors, which are important for bone repair, as well as several cytokines and proteins that are required for bone remodeling. More interestingly, ASCs possess the ability to release plasma membrane-derived vesicles (MVs), that are able to transfer different growth factors to neighboring cells by exerting pro-angiogenic and osteogenic effects during the regenerative process, and, therefore, ASCs may play an important regulatory role in bone tissue regeneration [9].

Moreover, the secretome of ASCs includes several endocrine and pro-angiogenic factors that can induce bone activity. With regards to bone tissue engineering, several researchers have demonstrated that BMSCs have a higher osteogenic differentiation capacity, compared to ASCs; whereas other scientists have shown that ASCs possess similar in vivo osteogenic potential, with respect to MSCs derived from bone marrow, with no sign of atypical proliferation [50,51,52,53].

Molecular investigations clearly confirmed how ERK, TNF-α, Notch, p38 MAPK, TGF-β, and JNK signaling pathways are strongly implicated in the osteogenic differentiation of ASCs.

It is important to note that the ability of ASCs to differentiate toward osteogenic lineage without any external stimulation, when they are placed on osteoconductive scaffolds during in vivo studies, makes these cells the most promising and usable candidates for translational regenerative therapy of craniomaxillofacial defects.

However, to strengthen the osteogenic commitment of ASCs, clinicians should combine such cells with osteoinductive scaffolds and bioactive molecules, such as BMP-2 [54,55].

As is well known to implant surgeons, oxygen and nutrients are critical for bone graft survival [52]. ASCs-based constructs should be implanted into a highly-vascularized surgical bed, even if the ability shown by ASCs to induce vessel formation is certainly a useful aid for new and proper bone growth. The combination of ASCs with scaffolds composed of natural polymers (chitosan and collagen), synthetic polymers (PLGA and polycaprolactone), as well as calcium phosphate-based ceramic scaffolds (hydroxyapatite and β-TCP), loaded with bioactive molecules, such as VEGF and BMP-2, seems to be the most promising strategy for the regeneration of bone defects in the craniomaxillofacial region [56]. In addition, ASC-derived microvesicles (MVs) containing osteogenic and pro-angiogenic molecules could also be enhanced through the use of a magnetic field [9]. Thus, MVs derived from ASCs cultured in a proper magnetic field could represent a new cell-based treatment to repair bone defects in the craniofacial region.

This review has clearly emphasized how clinical outcomes are strictly dependent on molecular mechanisms, and are often improved by a proper cell type to induce tissue formation. Correct analysis of the molecular and cellular phenomena is the only method of ensuring a realistic evaluation of clinical results using the scientific method.

## Figures and Tables

**Table 1:** Published Case Reports Using adipose-derived stromal/stem cells (ASCs) in Craniomaxillofacial Bone Regeneration.

Ref.	Study	Year	Disease	Scaffolds	Affiliation	Results	Authors
[14]	Autologous stem cells (adipose) and fibrin glue used to treat widespread traumatic calvarial defects: case report	2004	Calvarial fractures	Milled Bone from iliac crest with autologous Fibrin glue	Germany Justus-Liebig-University Medical School, Giessen	New bone formation and complete calvarial continuity after reconstruction	Lendeckel, S.; Jodicke, A.; Christophis, P.; Heidinger, K.; Wolff, J.; Fraser, J.K.; Hedrick, M.H.; Berthold, L.; Howaldt, H.P.
[15]	Novel maxillary reconstruction with ectopic bone formation by GMP adipose-derived stem cells	2009	Hemimaxillectomy due to a large keratocyst	β-TCP and BMP-2	Finland University of Tampere Helsinki University	Production of ectopic bone using auto ASC	Mesimaki, K.; Lindroos, B.; Tornwall, J.; Mauno, J.; Lindqvist, C.; Kontio, R.; Miettinen, S.; Suuronen, R.
[7]	Cranioplasty with adipose-derived stem cells and biomaterial: a novel method for cranial reconstruction	2011	Cranial defect	β-TCP granules	Finland Tampere University Hospital	Satisfactory outcomes in ossification	Thesleff, T.; Lehtimaki, K.; Niskakangas, T.; Mannerstrom, B.; Miettinen, S.; Suuronen, R.; Ohman, J.
[16]	Tissue engineering of bone: Clinical observations with adipose-derived stem cells, resorbable scaffolds, and growth factors	2012	Large craniofacial osseous defects	Resorbable scaffolds combined with rhBMP-2	Finland University of Tampere	Successful reconstruction of jaws, expect 3 failures	Sandor, G.K.
[19]	Adipose-derived stem cell (ASC) tissue engineered construct used to treat large anterior mandibular defect: A case report and review of the clinical application of GMP-level ASCs for bone regeneration	2013	Large anterior mandibular defects (left after tumour excision)	Titanium mesh filled with β-TCP granules and BMP-2	Finland University of Tampere University of Oulu Central Hospital of Central Finland Health Care District, Jyvaskyla, Finland	Mandibular reconstruction using the approach of in situ ossification with GMP-level ASCs	Sandor, G.K.; Tuovinen, V.J.; Wolff, J.; Patrikoski, M.; Jokinen, J.; Nieminen, E.; Mannerstrom, B.; Lappalainen, O.P.; Seppanen, R.; Miettinen, S.
[18]	GMP-level adipose-derived stem cells combined with computer-aided manufacturing to reconstruct mandibular ameloblastoma resection defects: Experience with 3 cases	2013	Three mandibular ameloblastoma resection defects	Β-TCP granules	Finland University of Tampere University of Oulu Central Hospital of Central Finland Health Care District, Jyvaskyla, Finland	Reconstruction of the three mandibular defects	Wolff, J.; Sandor, G.K.; Miettinen, A.; Tuovinen, V.J.; Mannerstrom, B.; Patrikoski, M.; Miettinen, S.
[17]	Adipose-derived Stem Cells Used to Reconstruct 13 Cases with Cranio-Maxillofacial Hard-Tissue Defects	2014	Cranio-maxillofacial defects: frontal sinus (3 cases); cranial bone (5 cases); mandible (3 cases); nasal septum (2 cases)	Bioactive glass granules (BAG); β-TCP granules; β-TCP strips; BMP-2	Finland University of Tampere University of Oulu Central Hospital of Central Finland Health Care District, Jyvaskyla, Finland	Successful integration of the construct to the surrounding skeleton (10/13 cases)	Sandor, G.K.; Numminen, J.; Wolff, J.; Thesleff, T.; Miettinen, A.; Tuovinen, V.J.; Mannerstrom, B.; Patrikoski, M.; Seppanen, R.; Miettinen, S.; et al.

**Table 2:** Registered Therapeutic Clinical Trials Using ASCs to Repair Maxillofacial Bone Defects (www.clinicaltrials.gov).

No.	Clinical Trials	Clinical Trials Number (Clinicaltrials.gov)	Disease/Condition	Scaffolds	Phase	Status	Affiliation	ASC-Administration	Enrolment
1	Filling Bone Defects/Voids with Autologous BonoFill For Maxillofacial Bone Regeneration	NCT02153268 link to clincialtrials.gov	Grafting after removal of cysts from jaws	OraGraft^®^ mineral particles (Bonofill)	I/II	Completed	Kfar Saba, Israel	Autologous ASCs combined with OraGraft^®^ mineral particles (Bonofill) Implantation of BonoFill to the maxillary or mandible defect/void	20
2	Filling Bone Defects/Voids With Autologous BonoFill-II for Maxillofacial Bone Regeneration	NCT02842619 link to clinicaltrials.gov	(1) Bone augmentation (Sinus augmentation) (2) Bone grafting after removal of cysts from jaws	OraGraft^®^ mineral particles (Bonofill)	I/II	This study is currently recruiting participants	Kfar Saba, Israel (Oral and Maxillofacial Surgery Clinic—Beit Merik)	Autologous ASCs combined with OraGraft^®^ mineral particles (Bonofill) Transplantation of BonoFill-II to the maxillary or mandible defect/void	20

## Clinical Trials

1. US National Library of Medicine: ClinicalTrials.gov. Available online: https://clinicaltrials.gov/ct2/show/NCT02153268?term=NCT02153268&rank=1 (accessed on 3 July 2017)
2. US National Library of Medicine: ClinicalTrials.gov: Available online: https://clinicaltrials.gov/ct2/show/NCT02842619?term=NCT02842619&rank=1 (accessed on 3 July 2017)

## References

[1] Bauer T.W., Muschler G.F. (2000). Bone graft materials: An overview of the basic science. Clin. Orthop. Relat. Res..

[2] De Long W.G., Einhorn T.A., Koval K., McKee M., Smith W., Sanders R., Watson T. (2007). Bone grafts and bone graft substitutes in orthopaedic trauma surgery: A critical analysis. J. Bone Jt. Surg. Am..

[3] Polo-Corrales L., Latorre-Esteves M., Ramirez-Vick J.E. (2014). Scaffold design for bone regeneration. J. Nanosci. Nanotechnol..

[4] Smith E., Kanczler J., Gothard D., Roberts C., Wells J., White L., Qutachi O., Sawkins M., Peto H., Rashidi H. (2014). Evaluation of skeletal tissue repair, part 1: Assessment of novel growth-factor-releasing hydrogels in an ex vivo chick femur defect model. Acta Biomater..

[5] Barba M., Cicione C., Bernardini C., Michetti F., Lattanzi W. (2013). Adipose-derived mesenchymal cells for bone regereneration: State of the art. BioMed Res. Int..

[6] Morcos M.W., Al-Jallad H., Hamdy R. (2015). Comprehensive review of adipose stem cells and their implication in distraction osteogenesis and bone regeneration. BioMed Res. Int..

[7] Thesleff T., Lehtimaki K., Niskakangas T., Mannerstrom B., Miettinen S., Suuronen R., Ohman J. (2011). Cranioplasty with adipose-derived stem cells and biomaterial: A novel method for cranial reconstruction. Neurosurgery.

[8] De Francesco F., Ricci G., D’Andrea F., Nicoletti G.F., Ferraro G.A. (2015). Human adipose stem cells: From bench to bedside. Tissue Eng. Part B Rev..

[9] Marędziak M., Marycz K., Lewandowski D., Siudzińska A., Śmieszek A. (2015). Static magnetic field enhances synthesis and secretion of membrane-derived microvesicles (MVs) rich in VEGF and BMP-2 in equine adipose-derived stromal cells (EqASCs)—A new approach in veterinary regenerative medicine. In Vitro Cell. Dev. Biol. Anim..

[10] Kang T., Jones T.M., Naddell C., Bacanamwo M., Calvert J.W., Thompson W.E., Bond V.C., Chen Y.E., Liu D. (2016). Adipose-derived stem cells induce angiogenesis via microvesicle transport of miRNA-31. Stem Cells Transl. Med..

[11] Alvira-Gonzalez J., Sanchez-Garces M.A., Cairo J.R., Del Pozo M.R., Sanchez C.M., Gay-Escoda C. (2016). Assessment of bone regeneration using adipose-derived stem cells in critical-size alveolar ridge defects: An experimental study in a dog model. Int. J. Oral Maxillofac. Implants.

[12] Sacak B., Certel F., Akdeniz Z.D., Karademir B., Ercan F., Ozkan N., Akpinar I.N., Celebiler O. (2017). Repair of critical size defects using bioactive glass seeded with adipose-derived mesenchymal stem cells. J. Biomed. Mater. Res. B Appl. Biomater..

[13] Tajima S., Tobita M., Orbay H., Hyakusoku H., Mizuno H. (2015). Direct and indirect effects of a combination of adipose-derived stem cells and platelet-rich plasma on bone regeneration. Tissue Eng. Part A.

[14] Lendeckel S., Jodicke A., Christophis P., Heidinger K., Wolff J., Fraser J.K., Hedrick M.H., Berthold L., Howaldt H.P. (2004). Autologous stem cells (adipose) and fibrin glue used to treat widespread traumatic calvarial defects: Case report. J. Craniomaxillofac. Surg..

[15] Mesimaki K., Lindroos B., Tornwall J., Mauno J., Lindqvist C., Kontio R., Miettinen S., Suuronen R. (2009). Novel maxillary reconstruction with ectopic bone formation by GMP adipose stem cells. Int. J. Oral Maxillofac. Surg..

[16] Sandor G.K. (2012). Tissue engineering of bone: Clinical observations with adipose-derived stem cells, resorbable scaffolds, and growth factors. Ann. Maxillofac. Surg..

[17] Sandor G.K., Numminen J., Wolff J., Thesleff T., Miettinen A., Tuovinen V.J., Mannerstrom B., Patrikoski M., Seppanen R., Miettinen S. (2014). Adipose stem cells used to reconstruct 13 cases with cranio-maxillofacial hard-tissue defects. Stem Cells Transl. Med..

[18] Wolff J., Sandor G.K., Miettinen A., Tuovinen V.J., Mannerstrom B., Patrikoski M., Miettinen S. (2013). GMP-level adipose stem cells combined with computer-aided manufacturing to reconstruct mandibular ameloblastoma resection defects: Experience with three cases. Ann. Maxillofac. Surg..

[19] Sandor G.K., Tuovinen V.J., Wolff J., Patrikoski M., Jokinen J., Nieminen E., Mannerstrom B., Lappalainen O.P., Seppanen R., Miettinen S. (2013). Adipose stem cell tissue-engineered construct used to treat large anterior mandibular defect: A case report and review of the clinical application of good manufacturing practice-level adipose stem cells for bone regeneration. J. Oral Maxillofac. Surg..

[20] Marędziak M., Marycz K., Tomaszewski K.A., Kornicka K., Henry B.M. (2016). The influence of aging on the regenerative potential of human adipose derived mesenchymal stem cells. Stem Cells Int..

[21] Lee K., Kim H., Kim J.M., Kim J.R., Kim K.J., Kim Y.J., Park S.I., Jeong J.H., Moon Y.m., Lim H.S. (2011). Systemic transplantation of human adipose-derived stem cells stimulates bone repair by promoting osteoblast and osteoclast function. J. Cell. Mol. Med..

[22] Cao L., Wang J., Hou J., Xing W., Liu C. (2014). Vascularization and bone regeneration in a critical sized defect using 2-*N*, 6-*O*-sulfated chitosan nanoparticles incorporating BMP-2. Biomaterials.

[23] Marenzana M., Arnett T.R. (2013). The key role of the blood supply to bone. Bone Res..

[24] Rehman J., Traktuev D., Li J., Merfeld-Clauss S., Temm-Grove C.J., Bovenkerk J.E., Pell C.L., Johnstone B.H., Considine R.V., March K.L. (2004). Secretion of angiogenic and antiapoptotic factors by human adipose stromal cells. Circulation.

[25] Kim Y., Kim H., Cho H., Bae Y., Suh K., Jung J. (2007). Direct comparison of human mesenchymal stem cells derived from adipose tissues and bone marrow in mediating neovascularization in response to vascular ischemia. Cell. Physiol. Biochem..

[26] Hsiao S.T., Asgari A., Lokmic Z., Sinclair R., Dusting G.J., Lim S.Y., Dilley R.J. (2012). Comparative analysis of paracrine factor expression in human adult mesenchymal stem cells derived from bone marrow, adipose, and dermal tissue. Stem Cells Dev..

[27] Peng H., Usas A., Olshanski A., Ho A.M., Gearhart B., Cooper G.M., Huard J. (2005). VEGF improves, whereas sFlt1 inhibits, BMP2-induced bone formation and bone healing through modulation of angiogenesis. J. Bone Miner. Res..

[28] Rosen V. (2009). BMP2 signaling in bone development and repair. Cytokine Growth Factor Rev..

[29] Biancone L., Bruno S., Deregibus M.C., Tetta C., Camussi G. (2012). Therapeutic potential of mesenchymal stem cell-derived microvesicles. Nephrol. Dial. Transplant..

[30] Pizzute T., Lynch K., Pei M. (2015). Impact of tissue-specific stem cells on lineage-specific differentiation: A focus on the musculoskeletal system. Stem Cell Rev..

[31] Vishnubalaji R., Al-Nbaheen M., Kadalmani B., Aldahmash A., Ramesh T. (2012). Comparative investigation of the differentiation capability of bone-marrow- and adipose-derived mesenchymal stem cells by qualitative and quantitative analysis. Cell Tissue Res..

[32] Monaco E., Bionaz M., Rodriguez-Zas S., Hurley W.L., Wheeler M.B. (2012). Transcriptomics comparison between porcine adipose and bone marrow mesenchymal stem cells during in vitro osteogenic and adipogenic differentiation. PLoS ONE.

[33] Shafiee A., Seyedjafari E., Soleimani M., Ahmadbeigi N., Dinarvand P., Ghaemi N. (2011). A comparison between osteogenic differentiation of human unrestricted somatic stem cells and mesenchymal stem cells from bone marrow and adipose tissue. Biotechnol. Lett..

[34] Follmar K., Decroos F., Prichard H., Wang H., Erdmann D., Olbrich K. (2006). Effects of glutamine, glucose, and oxygen concentration on the metabolism and proliferation of rabbit adipose-derived stem cells. Tissue Eng..

[35] Thangarajah H., Vial I.N., Chang E., El-Ftesi S., Januszyk M., Chang E.I., Paterno J., Neofytou E., Longaker M.T., Gurtner G.C. (2009). IFATS collection: Adipose stromal cells adopt a proangiogenic phenotype under the influence of hypoxia. Stem Cells.

[36] Hankenson K.D., Dishowitz M., Gray C., Schenker M. (2011). Angiogenesis in bone regeneration. Injury.

[37] Liu Q., Cen L., Zhou H., Yin S., Liu G., Liu W., Cao Y., Cui L. (2009). The role of the extracellular signal-related kinase signaling pathway in osteogenic differentiation of human adipose-derived stem cells and in adipogenic transition initiated by dexamethasone. Tissue Eng. Part A.

[38] Cho H.H., Shin K.K., Kim Y.J., Song J.S., Kim J.M., Bae Y.C., Kim C.D., Jung J.S. (2010). NF-κB activation stimulates osteogenic differentiation of mesenchymal stem cells derived from human adipose tissue by increasing TAZ expression. J. Cell. Physiol..

[39] Hess K., Ushmorov A., Fiedler J., Brenner R.E., Wirth T. (2009). TNFα promotes osteogenic differentiation of human mesenchymal stem cells by triggering the NF-κB signaling pathway. Bone.

[40] Lough D.M., Chambers C., Germann G., Bueno R., Reichensperger J., Swanson E., Dyer M., Cox L., Harrison C., Neumeister M.W. (2016). Regulation of ADSC osteoinductive potential using notch pathway inhibition and gene rescue: A potential on/off switch for clinical applications in bone formation and reconstructive efforts. Plast. Reconstr. Surg..

[41] Liu Y., Zheng W., Gao W., Shen Y., Ding W. (2013). Function of TGF-beta and p38 MAKP signaling pathway in osteoblast differentiation from rat adipose-derived stem cells. Eur. Rev. Med. Pharmacol. Sci..

[42] Samartzis D., Khanna N., Shen F.H., An H.S. (2005). Update on bone morphogenetic proteins and their application in spine surgery1. J. Am. Coll. Surg..

[43] Prockop D. (2007). “Stemness” does not explain the repair of many tissues by mesenchymal stem/multipotent stromal cells (MSCs). Clin. Pharmacol. Ther..

[44] Aulino P., Costa A., Chiaravalloti E., Perniconi B., Adamo S., Coletti D., Marrelli M., Tatullo M., Teodori L. (2015). Muscle extracellular matrix scaffold is a multipotent environment. Int. J. Med. Sci..

[45] Tatullo M., Falisi G., Amantea M., Rastelli C., Paduano F., Marrelli M. (2015). Dental pulp stem cells and human periapical cyst mesenchymal stem cells in bone tissue regeneration: Comparison of basal and osteogenic differentiated gene expression of a newly discovered mesenchymal stem cell lineage. J. Biol. Regul. Homeost. Agents.

[46] Marrelli M., Paduano F., Tatullo M. (2015). Human periapical cyst–mesenchymal stem cells differentiate into neuronal cells. J. Dent. Res..

[47] Paduano F., Marrelli M., White L.J., Shakesheff K.M., Tatullo M. (2016). Odontogenic differentiation of human dental pulp stem cells on hydrogel scaffolds derived from decellularized bone extracellular matrix and collagen type I. PLoS ONE.

[48] Paduano F., Marrelli M., Alom N., Amer M., White L.J., Shakesheff K.M., Tatullo M. (2017). Decellularized bone extracellular matrix and human dental pulp stem cells as a construct for bone regeneration. J. Biomater. Sci. Polym. Ed..

[49] Tobita M., Orbay H., Mizuno H. (2011). Adipose-derived stem cells: Current findings and future perspectives. Discov. Med..

[50] De Ugarte D.A., Morizono K., Elbarbary A., Alfonso Z., Zuk P.A., Zhu M., Dragoo J.L., Ashjian P., Thomas B., Benhaim P. (2003). Comparison of multi-lineage cells from human adipose tissue and bone marrow. Cells Tissues Organs.

[51] Cowan C.M., Shi Y.Y., Aalami O.O., Chou Y.F., Mari C., Thomas R., Quarto N., Contag C.H., Wu B., Longaker M.T. (2004). Adipose-derived adult stromal cells heal critical-size mouse calvarial defects. Nat. Biotechnol..

[52] Grayson W.L., Bunnell B.A., Martin E., Frazier T., Hung B.P., Gimble J.M. (2015). Stromal cells and stem cells in clinical bone regeneration. Nat. Rev. Endocrinol..

[53] Tatullo M., Marrelli M., Amantea M., Paduano F., Santacroce L., Gentile S., Scacco S. (2015). Bioimpedance detection of oral lichen planus used as preneoplastic model. J. Cancer.

[54] Marrelli M., Tatullo M. (2013). Influence of PRF in the healing of bone and gingival tissues. Clinical and histological evaluations. Eur. Rev. Med. Pharmacol. Sci..

[55] Perniconi B., Coletti D., Aulino P., Costa A., Aprile P., Santacroce L., Chiaravalloti E., Coquelin L., Chevallier N., Teodori L. (2014). Muscle acellular scaffold as a biomaterial: Effects on C2C12 cell differentiation and interaction with the murine host environment. Front. Physiol..

[56] Mele L., Vitiello P.P., Tirino V., Paino F., De Rosa A., Liccardo D., Papaccio G., Desiderio V. (2016). Changing paradigms in cranio-facial regeneration: Current and new strategies for the activation of endogenous stem cells. Front. Physiol..

